# Cryosurgery in oral lesions

**DOI:** 10.4317/jced.63133

**Published:** 2025-10-01

**Authors:** Eleni Georgakopoulou, Panagiota Loumou, Dimitrios Sgouros, Akhilanand Chaurasia, Antonios Panagiotopoulos

**Affiliations:** 1 MD, DDS, MSc, PhD. Private Practice, 4 Fokaias St, Athens 14232, Greece; 2 MD, DDS, MSc, PhD. Molecular Carcinogenesis Group, Medical School, National and Kapodistrian University of Athens, Greece; 3 MD, DDS, PhD. Private Practice, 44 Nefelis St, Athens, Greece; 4 MD, PhD. 2nd Department of Dermatology and Venereology, Attikon General University Hospital, Medical School, National and Kapodistrian University of Athens, Rimini 1, Chaidari 12462, Athens, Greece; 5 MDS, Department of Oral Medicine and Radiology, King George’s Medical University, India; 6 MD. Former Head of the Cryosurgery Department, Hospital “A. Syggros,” Athens, Greece; 7 MD. Dermatologist-Venereologist, Private Practice, 36 Zoodochou Pigis St, Athens 10681, Greece

## Abstract

Cryosurgery is a treatment method that employs extremely low temperatures to address various diseases. Since the early 1960s, liquid nitrogen has been utilized in dermatology, oral surgery, ophthalmology, gynecology, urology, cardiology, and internal organ surgery. It is the preferred treatment for numerous skin conditions due to its versatility in addressing benign, precancerous, and malignant diseases.

The application of cryosurgery as a therapeutic approach for oral mucosal diseases has been shaped by the treatment of analogous dermatological conditions. Considering the potential of cryosurgery applications, it has been utilized as an alternative therapy for various oral lesions.

** Key words:**Cryosurgery, Oral lesions, Liquid nitrogen, Oral mucosal diseases, Cryotherapy mechanisms, Cryogens, Oral medicine.

## 1. Introduction

The term Cryosurgery is derived from the Greek words “Cryo” (cold) and “Cheirourgiki” (surgery). Cryosurgery is characterized by the intentional and regulated destruction of pathological tissue through the application of extremely low temperatures [1].

The initial documentation of utilizing cold therapy for medical conditions originates from ancient Egypt. Hippocrates also documented the application of cold to alleviate swelling, pain, and hemorrhage in his writings [2]. James Arnott (1797-1883), an English physician, was the first who used controlled freezing to treat surface lesions of the skin and the palliation of cancerous tumors [3].

The most notable advancement in cryosurgery in contemporary times was the invention by Irving Cooper, an American neurosurgeon. He designed the first cryosurgical apparatus, using liquid nitrogen in a closed circuit [4].

Currently, cryosurgery is extensively employed by dermatologists and stomatologists to address various skin and oral ailments, either as a primary treatment or as an alternative because of its simplicity, efficacy, and cost-effectiveness. S. Zacarian conducted numerous experimental studies using the oral mucosa of hamsters [1].

Our current review aims to describe and explore the potential and scope of cryosurgical techniques, as well as to summarize their applications in oral diseases.

## 2. Mechanism of action

Cooling is defined as the removal of heat. Heat transfer consistently occurs from the warmer object to the cooler one. When a cold object contacts the skin’s surface, it extracts heat from the skin, resulting in a tissue reaction [5]. The impact of cold on human tissue is contingent upon the rate of temperature decline, duration of freezing, duration of thawing, solute concentration, and the lowest temperature attained within the tissue. For complete destruction of benign lesions, a temperature of -200 to -30°C is required, while for effective eradication of malignancies, a temperature of -400 to -50°C is required [6,7].

Slow cooling produces extracellular ice, while rapid cooling produces intracellular ice. So rapid cooling is desirable, causing irreversible damage to cell- membranes, mitochondria, and endoplasmic reticulum [5]. Slow thaw times and repeat freeze-thaw cycles produce further tissue injury. An increase in solute concentrations inside the cell occurs due to osmolarity changes, which disrupt cell membranes [8].

Freezing induces vasoconstriction and decreases blood supply, subsequently leading to vasodilation [9]. Later, within 30–40 minutes, vascular stasis develops, followed by endothelial damage, increased vascular permeability, platelet aggregation, and the formation of microthrombi [9]. The formation of microthrombi results in ischemic necrosis and resolution of pathological tissue [9].

## 3. Cryogens

Cryogens are materials used to perform cryotherapy; however, the type of cryogens needed to treat dermatological lesions or oral lesions depends on the type of lesions to be treated, the frequency of its use, and the physician’s expertise in the method [10,11].

The most commonly used cryogens are ( Table 1).

3.1 LIQUID NITROGEN (LN2)

Liquid nitrogen is the most effective cryogen for clinical use. With liquid nitrogen a temperature of -25oC to -50oC can be achieved within 30 seconds [16].

3.2 NITROUS OXIDE (N2O) 

Nitrous oxide is a nonflammable gas with a boiling point of -89oC [17]]. Ιt is readily available, storable, and easily applied either through a probe or as a spray. N2O should be reserved only for benign lesions [17].

N2O can be used in any area of the oral cavity due to the suitability of the cryoprobes available for the various devices. The contact cryoprobe of the N2O thaws very quickly as soon as the application of cooling stops and is easily detached from the mucous membranes without tearing them. It does not need pre-cooling, at least in most devices, unlike the LN2.

3.3 SOLIDIFIED CARBON DIOXIDE

It is used in two forms: solid (dry ice) and gas. In both forms it produces a probe temperature of -78°C [18].

3.4 HISTOFREEZER

Histo-freezer is a cryogen that uses a safe mixture of dimethyl ether and propane. The spraying of these gases through an applicator onto a foam bud can produce a temperature of -75°C [19].

## 4. Equipment

4.1. Equipment for e

The basic equipment for routine cryosurgery (LN) is listed in  Table 2 Table 2 [11,12,14,20].

For liquid nitrogen:

● A Dewar storage tank with a capacity of 25–30 liters is required. Dewar gas containers are metal cylinders designed to store cryogens as compressed gas. They feature an inbuilt internal pressure equalization mechanism and are available in various sizes, ranging from 5 liters to 50 liters [14,21].

● Handheld units are the most used instruments in cryosurgery. These lightweight, small metal vacuum bottles are equipped with a controllable trigger to start and stop the freezing process [18,22,23].

● Spray tips are metal attachments that screw onto handheld units. These tips come with round apertures ranging from size A (0.04 inches) to size E (0.016 inches). Bent spray tips are also available for enhanced versatility [21].

● Open cones are conical devices that are open at the base and apex. They concentrate liquid nitrogen in a specific area, enabling faster and deeper freezing while protecting sensitive regions, such as the eyes. Otoscopes can also be used for this purpose [13,20,24].

Probes: Cryoprobes are metal discs through which liquid nitrogen is circulated. Cryoprobes come in all shapes and sizes. They come in various shapes and sizes and are particularly useful for treating vascular or cystic lesions, such as angiomas, mucoceles, and venous lakes. Cryoprobes compress and freeze the lesion simultaneously [20,21,25].

Cryo-tweezers: Specially designed tweezers and forceps are used to treat protruding skin lesions, such as skin tags. These tools enable precise and controlled freezing of the lesion [26].

4. 2 Equipment for N2O

The freezing medium, nitrous oxide (N2O), is stored under high pressure in its liquid form within a steel cylinder.

This cylinder is equipped with special accessories, such as a cryoprobe and cryoprobe spray, both of which are cooled via the Joule-Thomson effect. Additionally, a single-use cotton swab can be utilized with the cryoprobe spray.

## 5. Techniques

5.1. TECHNIQUES FOR LN2.

There are six common cryosurgical techniques ( Table 3).

5.1.1 Dip stick technique.

This is a simple method. A cotton bud is dipped into liquid nitrogen and firmly applied to the lesion until a halo of ice forms around the bud. For larger lesions, repeated applications may be necessary to achieve sufficient deep freezing [13,14,27].

5.1.2 Open spray technique

**Figure 1:**
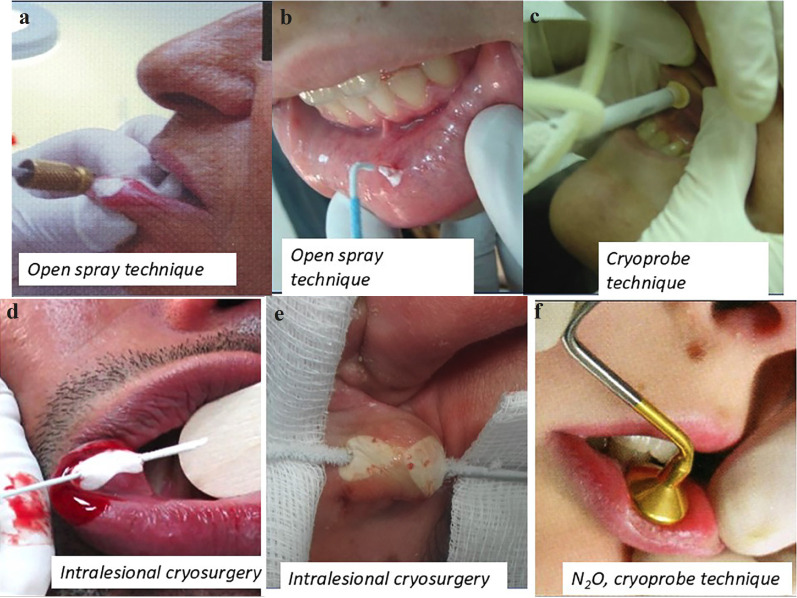
Cryosurgery techniques.

This technique is highly suitable for physicians who perform cryosurgery, as it maximizes lesion destruction while minimizing morbidity. In the spray technique, the liquid nitrogen spray tip is held approximately 1 to 1.5 cm from the mucous membrane, positioned over the center of the area to be treated [12]. The lateral spread of freezing typically extends 1–2 mm beyond the visible pathological border for benign lesions and up to 5 mm for malignant lesions [13,14,28] (Fig. 1a,b).

5.1.3 Cryoprobe Technique

Also known as contact technique, this method involves applying a precooled metal disc directly to the lesion. In this technique , liquid nitrogen is circulated through the cryoprobe to cool the metal disc before application to the lesion. Cryoprobes are available in various sizes and types, making them versatile for different clinical needs. They are particularly effective in treating vascular lesions, as the pressure exerted by the cryoprobe displaces blood from the tissues, enabling more effective treatment [13,14,28] (Fig. 1c).

PEARLS FOR LIQUID NITROGEN PROBES 

● Pre-freeze the tip of the probe before applying it to the mucosa.

● If the probe sticks to the patient’s tissue, avoid pulling it off forcibly 

● If a probe of the exact size is unavailable, it is preferable to use a smaller probe and extend the freezing duration [21].

5.1.4 Tweezers

The proper technique for freezing protruding lesions is to freeze only the fleshy part protruding from the surface of the skin or the mucous, taking care not to extend the ice formation to surrounding base tissue [26].

5.1.5 Intralesional cryosurgery 

The intralesional technique involves inserting a thick needle longitudinally through the center of a tumor. The needle is connected to the cryosurgery unit via a Luer-lock adapter. Cryogen is then delivered through the needle, freezing the lesion from the inside out [29] (Fig. 1d,e).

5.2 TECHNIQUES FOR N2O 

N2O techniques utilize the Joule-Thomson effect, which is based upon the adiabatic principle—the cooling effect achieved by expanding gas through a small opening. In cryoprobes, gas is delivered at high pressure through a small nozzle or annulus at the probe tip. As the gas exits this restricted orifice, its pressure drops rapidly from 50 kg/cm2 (700 lb/in2) to 1.4 kg/cm2 (20 lb/in2). The expansion results in cooling, lowering the temperatures to approximately -70°C. The cooled gas lowers the external tip temperature of the cryoprobe, freezing it to a range of -40°C to -70°C, depending on the design of the cryoprobe [30].

There are two main techniques for N2O cryosurgery:

5.2.1 The cryoprobe technique

N2O, due to its smaller cryogenic effect compared to LN, causes fewer side effects, such as pain or hypo- or hyperpigmentation. For this reason, it is a preferred choice for treating lesions on the lips [31] (Fig. 1f).

5.2.2 The open spray technique 

This technique is applied similarly to the liquid nitrogen (LN) method [12,32].

## 6. Treatment protocol

6.1. PRE-OPERATIVE CARE

6.1.1. Counseling and consent

It is essential to thoroughly explain the procedure to the patient. This includes detailing the technique, expected results, post-treatment care, potential blister formation, and possible complications. A written informed consent must be obtained, ensuring the patient understands all aspects of the procedure [14].

6.1.2. History taking

A detailed medical history should be taken. Consider factors such as sensitivity to cold, cold urticaria, vascular insufficiency, Raynaud phenomenon, autoimmune diseases, and past treatments for the lesion. Document any recurrences, as instructed in references [14,33].

6.1.3. Physical examination 

A detailed physical examination must be performed focusing on the , size, location margin depth, of the lesion.

6.1.4 Biopsy is necessary for premalignant or malignant lesions or if the diagnosis is uncertain

6.1.5. Pre-operative preparation 

The area to be treated should be thoroughly cleaned with a mild antiseptic solution. If the patient is anxious, an analgesic or sedative should be administered. The surrounding healthy mucosa should be protected to prevent the spread of LN to unaffected areas. Intralesional anesthesia is not required for benign lesions but may be required for malignant lesions [14,34].

## 7. Treatment

Cryosurgery effectively treats benign and premalignant lesions of the oral cavity and compares favorably with other therapeutic methods. Like any other therapeutic procedure, cryosurgery has its advantages and disadvantages, as well as specific indications and contraindications ( Table 4, Table 5, Table 6).

The advantages of cryosurgery specifically in the oral cavity are summarized in  Table 5.

Each cryosurgery procedure should be record for legal, audit and educational purposes. The treatment protocol should include  Table 7.

7.1 CRYOSURGERY FOR BENIGN ORAL LESIONS

Cryosurgery is an effective treatment for benign lesions in the oral cavity, and it compares favorably to other therapeutic methods [35]. Benign lesions usually require superficial freezing. It is better to undertreat than overtreat, and if it is necessary, repeat. Local anesthesia usually is not required, ( Table 8).

7.1.1 HPV LESIONS

Verruca vulgaris is relatively uncommon in the oral mucosa. The oral lesions may be single or multiple and are typically located on the lips, palate, and , less frequently, in other oral areas.

Warts respond well to cryosurgery with a spray technique ( using LN2 or N2O). The entire lesion, including a small portion of the base and surrounding rim, is frozen [10,38].

**Figure 2:**
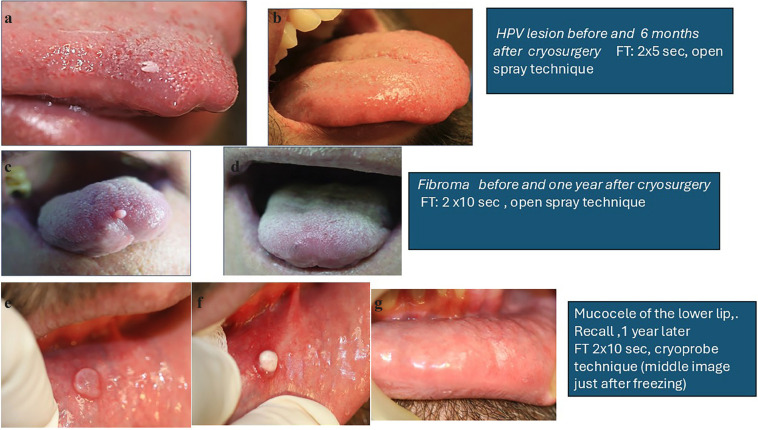
Cryosurgery applications.

Condyloma acuminatum in the oral mucosa is rarely encountered and may result from autoinoculation from genitals or during orogenital contact (Fig. 2a,b). Condylomata acuminata also respond favorably to cryosurgery. The freezing time varies from 5 to 15 seconds.

Focal epithelial hyperplasia, or Heck disease, is a rare oral mucosal condition caused by the HPV serotypes 13 and 32. It appears as a benign epithelial growth and responds favorably to cryosurgery. The lesions are frozen for 15 seconds, often in one or two sessions [39].

7.1.2 FIBROMA

Fibroma is the most common benign tumor of the oral cavity. The lesion typically presents as an asymptomatic, well-defined, firm, sessile, or pedunculated tumor with a smooth surface covered by normal epithelium [38]. The open spray technique is used for treatment, with a freezing time of 10-15 seconds (Fig. 2c,d).

7.1.3 MUCOCELE

Mucoceles are common lesions on the lower lip, typically presenting as soft, red or blue cysts up to 1 cm in diameter. These lesions, which originate from minor salivary glands, appear to be caused by epithelial proliferation of a partially obstructed salivary duct, preventing adequate drainage of saliva. This leads to duct dilation and swelling [40].

The lesion is first deroofed to evacuate the gelatinous material. The frozen probe is then pressed onto the lesion for about 10-30 seconds. No lateral spread of ice is necessary [13]. The dipstick technique can also be used with a freezing time of 10-30 seconds. Yeh *et al* treated 33 patients with this method for 30-50 seconds, applyinga second treatment, only if a residual lesion remained. There was recurrence in two patients which were also treated with cryosurgery to achieve complete resolution [38] (Fig. 2e,f,g).

7.1.4 PYOGENIC GRANULOMA

Pyogenic granuloma is a common, tumor-like granulation tissue overgrowth of the oral tissues in response to mild irritation [1] .

Cryosurgery can be used to treat pyogenic granuloma, although it is advisable to obtain tissue for histological examination. The open spray technique is used. Freezing time varies between 10 to 15 seconds [38]. Narula and Malik treated 3 pyogenic granulomas using nitrous oxide closed system with a freezing time of 2 minutes [37]. Two sessions were required for each lesion to achieve regression.

7.1.5 VENOUS LAKE

**Figure 3:**
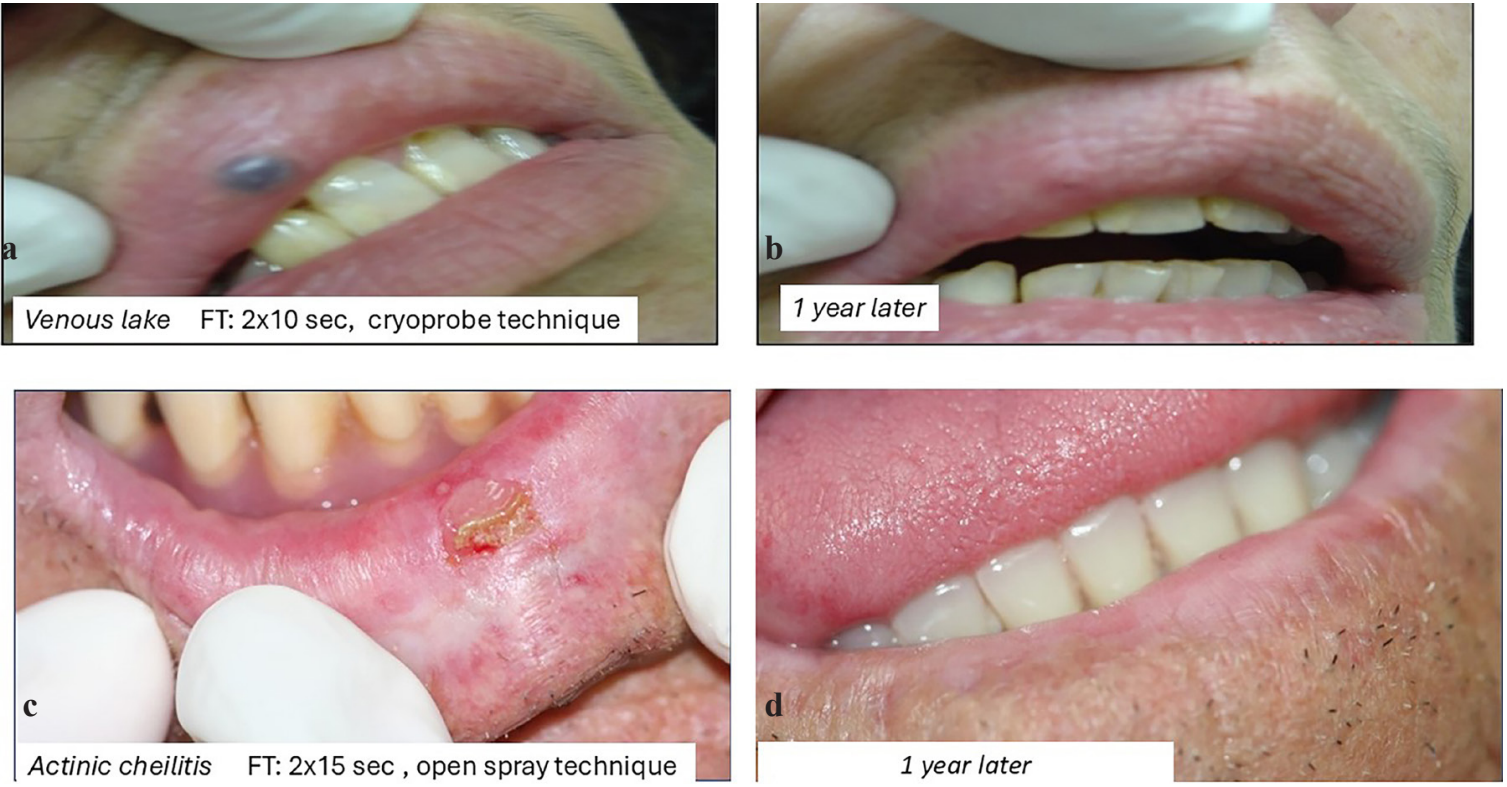
Cryosurgery applications 2.

Venous lake of the lips responds well to cryosurgery. The cryoprobe technique is used, yielding excellent cosmetic results. The probe is applied with firm pressure to the center of the lesion to expel the blood content. The freezing time varies between 15-30 seconds. Patients should be informed about the local edema, which may persist for a few days [10,38]. The cotton-tipped dipstick can also be used with one freeze-thaw cycle of 5-10 seconds. Cryosurgical treatment of venous lakes is safe, yields excellent results, is inexpensive and is suiTable for most patients [37,38] (Fig. 3a,b).

7.1.6. SMALL HEMANGIOMAS 

Small hemangiomas respond well to cryosurgery. The lesion is first emptied by compression with the probe before and during treatment [37,38] Freezing time varies between 10-20 seconds. Nogueira treated a 65 old woman with a 1.5 cm hemangioma on the left lateral border of the tongue using LN spray. The treatment consisted of a single session with two cycles of 1 minute each, resulting in excellent outcomes [22].

7.1.7 LYMPHANGIOMA 

Lymphangiomas are less responsive to cryosurgery. However, the lesions can be controlled through repeated applications. The probe must be firmly applied until a halo of 1-2 mm appears around the lesion [37,38].

7.1.8 ORAL MELANOTIC MACULE 

Oral melanotic macule refers to brown or black melanotic macules on the oral mucosa that are not linked to racial pigmentation or any systemic disease or syndrome [1]. These lesions respond favorably to cryosurgery. The open spray or cotton swab techniques are commonly used. A double freeze-thaw cycle with a 1 mm margin typically yields excellent results, usually in just one session [2,27].

7.1.9. FAMILIAL ACANTHOSIS NIGRICANS 

Cryosurgery is effective in the treatment of familial acanthosis nigricans. The open spray technique is used with a freezing time of 15-20 seconds [38].

7.1.10 MEDIAN LIP FISSURE 

Median lip fissure is a rare benign lesion characterized by linear ulceration in the middle of the lower lip [1]. Median lip fissures are often painful and may appear intermittently over a period of years, or they can be intracTable and persist for a long time. Cryosurgery is effective in the elimination of chronic lip fissure [31,37,38].

7.1.11 PALATAL PAPILLARY HYPERPLASIA 

Papillary hyperplasia of the palate is a form of denture stomatitis that occurs in patients who wear ill-fitting dentures for many years [1]. Patients have effectively treated papillary hyperplasia of the palate with cryosurgery, achieving very satisfactory results [37].

7.1.12 LAUGIER–HUNZINKER SYNDROME 

Laugier-Hunzinker syndrome is an acquired benign pigmentary skin condition that affects the oral cavity, including the lower lip, and presents as brown-black macules ranging from 1 to 5 mm in size [1,2]. It is frequently associated with longitudinal melanonychia. Cryosurgery has been used for the treatment of Laugier-Hunziker syndrome with favorable results [2,37].

7.1.13 LIPOID PROTEINOSIS 

Lipoid proteinosis is a rare hereditary metabolic disorder transmitted as an autosomal recessive trait. It is characterized by the deposition of an amorphous hyaline-like material (glycoprotein) in the mucous membranes, skin, and various internal organs [1]. AM Shirani treated a 24-year-old female with nodules using N2O for one minute [3]. Cryosurgery was performed in two sessions. After treatment, the lips were softer and had improved aesthetics. The use of cryosurgery offers advantages over surgery for reshaping lip lesions in this syndrome, as suturing is not feasible due to the rigidity of the mucosa in these patients [3].

7.2. CRYOSURGERY FOR PRECANCEROUS CONDITIONS

Cryosurgery in oral precancerous lesions 

Cryosurgery is employed in the treatment of the following precancerous conditions of the oral cavity:

- ACTINIC CHEILITIS

- LEUKOPLAKIA 

7.2.1. ACTINIC CHEILITIS 

Actinic cheilitis is a pre-invasive malignant lesion of the lips that is caused by prolonged exposure to solar radiation.

Actinic cheilitis can progress to squamous cell carcinoma (SCC), and SCC of the lip metastasizes more frequently than SCC of the skin. Therefore, early treatment of actinic cheilitis is crucial for prevention. Cryosurgery is an effective treatment for actinic cheilitis, using either the closed-probe or open-spray technique. Lubritz and Smolewski reported a cure rate of 96.2% for 53 lesions of the lower lip in 37 patients. The open-spray technique was employed, with thaw times ranging between 60 and 90 seconds. The freezing time with nitrogen spray can range from 10 to 15 seconds for areas up to 2 cm [6] (Fig. 3c,d).

7.2.2 LEUKOPLAKIA 

The World Health Organization (WHO) defines leukoplakia as “a white patch or plaque that cannot be characterized clinically or histopathologically as any other disease” [1,3,4]. It is crucial to monitor oral leukoplakia closely because of the risk of malignant transformation into oral squamous cell carcinoma (SCC), which can have severe consequences.

There are several treatment options for oral leukoplakia, including non-surgical and ablative procedures. Nonsurgical therapeutic options include vitamin A, retinoids, beta carotene, vitamin E, bleomycin alpha-tocopherol. Ablative procedures include surgical excision, application of CO2, photodynamic therapy and cryosurgery [4,6,7,9] Cryosurgery is an effective alternative treatment for oral leukoplakia, though biopsies should be performed on all lesions prior to treatment.

Cryosurgery can be carried out using the open spray technique, which involves directly applying the cryogen to the lesion [27], or the cryoprobe technique, which provides a greater degree of temperature control. A cotton swab technique can also can be used for benign and superficial lesions, where the swab is dipped into liquid nitrogen for 1-2 seconds and applied to the lesion with light pressure for 30 seconds. The number of freeze -thaw cycles depend on the area and type of lesion [27].

Cryosurgery for oral leukoplakia requires rapid freezing, slow thawing, 3-4 mm margins and two to three freezing -thawing cycles [6,7,9]. During the procedure, high power suction can be used to remove saliva and vapor fog to improve visibility 64.

Instead of liquid nitrogen, nitrous oxide can also be used as a cryogen. Varum Kumar treated 25 lesions in 19 patients. A single session was performed with two cycles of freezing for one and a half minutes followed by a 1-minute thaw. Complete regression was achieved in 22 of the lesions (88%), while 2 lesions (8%) required retreatment, resulting in complete regression [6,7,9].

7.3 CRYOSURGERY FOR MALIGNANT LESIONS 

Cryosurgery is generally not considered the treatment of choice for malignant neoplasms in the oral cavity [37]. However, it can still be valuable in certain situations, either as a complementary treatment or for palliative care in patients with cancer.

7.3.1 SQUAMOUS CELL CARCINOMA

The use of cryosurgery for the treatment of oral SCC is mainly palliative. It can be used on inoperable carcinoma or when relapses occur. Under certain conditions, it can be considered as an initial treatment in patients with inoperable neoplasms, in high-risk elderly patients, and advanced stage HIV-patients. A longer freezing time is required (7 minutes) [12]. Because there is a possibility of severe postoperative edema and bleeding, hospitalisation may be required. Combining cryosurgery with chemotherapy may offer additional benefits [12,37,38].

7.3.2 KAPOSI’s SARCOMA 

Kaposi’s sarcoma is highly responsive to cryosurgery, particularly the macular and maculopapular forms. Most lesions require only a single 15-30 s FTC, with a 3 mm margins of surrounding tissue. However, plaques and nodules tend to have a poor response. For these, the cryoprobe technique is recommended [14].

7.3.3 MELANOMA 

Cryosurgery of Malignant Melanoma is not indicated except in cases of inoperable melanomas for palliative reasons [37,38].

## 8. Complications

Complications can occur after a cryosurgical procedure. Complications can be divided into immediate, delayed, prolonged and permanent ( Table 9) [6,10,13,38,39].

## 9. Post treatment care

Written instructions must be provided after cryosurgical procedure to the patients. Patients must be informed about the edema, especially when treatment has been done to the lip [38].

Dear Sir/Madam, you have been treated with cryosurgery. Cryosurgery submitted using liquid nitrogen which cools the mucosa at very low temperatures to destroy the lesion. The following instructions will help you troubleshoot any problems that may occur:

● If you feel pain use an analgesic drug

● The first two days is better to eat cold and soft food

● If the damage is in the gums, you should not wash your teeth for a week

● If the damage is on the dermal junction use the antibiotic ointment, we provide for 7-10 days

● Use the solution we provide for 2 weeks

● If there is bleeding press the pleading point with a gauze impregnated with oxygenated solution

● If there is a large swelling then contact the treating physician

● Your follow up is in … days

## 10. Conclusions

By careful selections of patients Cryosurgery is a simple, easy, effective and well-tested method. It can constitute a useful alternative treatment for many benign and some precancerous lesions. Correct diagnosis and good knowledge of cryosurgical principles contribute greatly to the successful treatment.

## Figures and Tables

**Table 1:** Cryogens [6,11-15].

Liquid nitrogen LN2	-196°C
Nitrous oxide N2O	-89°C
Solidified Carbon Dioxide	-78°C
Chlorodifluoromethane	-41°C
Dimethyl ether and propane	-24°C, -42°C

**Table 2:** Equipment for cryosurgery LN2.

· Dewar storage tank
· Handheld cryosurgical unit
· Cryospray nozzles
· Set of cryoprobes
· Set of neoprene or otoscope cones
· Cryo-tweezers

**Table 3:** Techniques for LN2 Cryosurgery [13,14,27,28,29].

1.	Dip stick (cotton swab) technique
2.	Open spray technique
3.	Semi-open spray technique
4.	Close (probe) technique
5.	Tweezers
6.	Intralesional cryosurgery
Each one uses different equipment	

**Table 4:** General indications for cryosurgery [35,36].

· Patients of all ages, including children and elderly people whose health may be fragile
· Patients who have adverse reactions to anesthesia. Cryosurgery can be performed without anaesthesia
· Patients on anticoagulation treatment
· Patients with pacemakers
· Patients with infectious diseases
· Pregnant women
· Patients who fear surgical methods

**Table 5:** Advantages of Cryosurgery in the Oral Cavity [37,38].

· The oral cavity is easily accessible
· Method of small risk and cost
· Fast treatment at clinic site
· The moisture of the mouth and the soft tissue favours the rapid spread of ice ball
· Possibility of using a local cream for anaesthesia
· Ease of postoperative follow-up
· Minimum risk of postoperative bleeding
· Post-operative infections are rare
· Complications are rare
· Healing is good
· Cure rates are high

**Table 6:** Contraindications of cryosurgery in the oral cavity [28,39,40].

A. ABSOLUTE CONTRAINDICATIONS
1. Lesion for which tissue pathology is required 2. Proven sensitivity or adverse reaction to cryosurgery 3. Melanoma 4. SCC 5. Cold urticaria
B. RELATIVE CONTRAINDICATIONS
1. Cold intolerance 2. Collagen disease or autoimmune diseases 3. Concurrent treatment with immunosuppressive drugs 4. Cryoglobulinemia 5. Multiple myeloma 6. Pyoderma gangrenosum 7. Raynaud's disease

**Table 7:** Treatment protocol [38].

· Name
· Birthdate
· Address, telephone, e-mail
· Date of treatment
· Photo before
· Clinical diagnosis of lesion
· Location, size of lesion
· Histological diagnosis especially in precancerous and cancerous lesions
· Technique (cryoprobe, open spray, cotton-swab) exact procedures carried out (e.g. cryogen used, technique, freeze/thaw cycle) nozzle size, Freezing Time (FT) , Thaw Time (TT) (one or two freeze-thaw cycles)
· Any complications
· Follow-up (1 week, 2 weeks, 6 months, 1 year, 2 years, 5 years)
· Photo after

**Table 8:** Benign oral lesions amenable to cryosurgery [38,42,43].

TYPE OF LESION	TECHNIQUE	FT/N2	FT/N2O	MARGINS	INTERVALS
HPV	OS	10 sec	15 sec	1 mm	3 weeks
Fibroma	CP	10 sec	20 sec	2 mm	30 days
Mucocele	CP	15 sec	30 sec	2 mm	30 days
Pyogenic granuloma	OS	10 sec	20 sec	2 mm	3 weeks
Venous Lake	CP	10 sec	30 sec	1 mm	3 weeks
Palatal familial hyperplasia	OS	10 sec	10 sec	1 mm	30 days
Labial lentigines macules	OS	10 sec	10 sec	1 mm	2 weeks
Hemangiomas	CP	10 sec	20 sec	1 mm	3 weeks
Lymphangioma	CP	10 sec	20 sec	1 mm	3 weeks
Familial acanthosis nigricans	OS	15 sec	15 sec	1 mm	20 days
Median lip fissure	OS	15 sec	10 sec	1 mm	15 days
Oral lichen planus	OS	10 sec	20 sec	1 mm	15 days
Laugier-Hunziker syndrome	OS	15 sec	10 sec	1 mm	20 days
Lipoid proteinosis	OS	10 sec	10 sec	1 mm	15 days

**Table 9:** Complications of cryosurgery.

IMMEDIATE	Swallowing liquefied nitrogen Tearing of the mucosa due to violent detachment of the cryogen Pain Tooth pain Edema Hemorrhage Syncope Blister formation
DELAYED	Postoperative infection and febrile reaction Haemorrhage Granulation tissue formation
PROLOGNGED	Hyperpigmentation of the lips Nerve/nerve ending damage
PERMANENT	Hypopigmentation Tenting or notching of the vermillion border of the lip Atrophy Trismus Sialadenitis

## Data Availability

The datasets used and/or analyzed during the current study are available from the corresponding author.
